# Virtual Anatomical Sections of the Dromedary Head (*Camelus dromedarius*): A Descriptive 3D CT‐Based Study of Brain–Skull Relationships

**DOI:** 10.1155/vmi/3214441

**Published:** 2026-03-01

**Authors:** Bitsha-Kitime Dieudoné Kabkia, Lobna Ouertani, Germain Nissao Magnibo, Abdelmonem Ben Khalifa

**Affiliations:** ^1^ Anatomy, Histology and Embryology Department, Inter-State School of Sciences and Veterinary Medicine, Dakar, Senegal; ^2^ Anatomy Department, National School of Veterinary Medicine (ENMV), Sidi Thabet, Ariana, Tunisia

**Keywords:** 3D anatomy, brain, comparative neuroanatomy, dromedary, sinus, skull, virtual sections

## Abstract

The aim of this study was to provide a descriptive three‐dimensional (3D) computed tomography (CT)–based visualization of the spatial relationships between the brain and the skull in the dromedary camel (*Camelus dromedarius*), using virtual anatomical sections. The study was conducted on the head of a single 18‐month‐old male dromedary obtained postmortem following slaughter for human consumption. CT images were acquired and processed using 3D reconstruction techniques to generate axial, sagittal, and dorsal virtual sections. These reconstructions illustrate the organization of cranial bones, paranasal sinuses, major brain regions, and intracranial spaces, highlighting their spatial integration. Rather than aiming at morphometric or population‐based analysis, this atlas‐style approach focuses on anatomical topography and educational value. Although limited to a single specimen, the study provides a useful visual reference for veterinary anatomy teaching, comparative neuroanatomy, and clinical imaging interpretation in camelids.

## 1. Introduction

Understanding the topographical relationships between the brain and the skull is fundamental for veterinary neuroanatomy and comparative anatomical studies. Although these aspects are well documented in domestic species such as cattle, horses, and dogs, information remains limited for the dromedary camel (*Camelus dromedarius*), despite its remarkable physiological and anatomical adaptations to desert environments.

Advances in computed tomography (CT) and three‐dimensional (3D) reconstruction have significantly improved the ability to visualize complex anatomical structures without invasive dissection. 3D CT offers high‐resolution spatial representation of bones, soft tissues, sinuses, and neurovascular elements, making it a powerful tool for anatomical description and veterinary education.

Several studies have used CT, MRI, and cross‐sectional anatomy to document the camel head and other anatomical regions. Alsafy et al. [1] combined sectional anatomy with 3D CT to describe the heart ventricles of the dromedary. Alsafy et al. [2] provided a detailed CT and gross anatomical description of the camel head. Emam et al. [3] produced a comprehensive MRI/CT/cross‐sectional atlas of the normal camel head structures. More recently, Kabkia et al. [4] described the paranasal sinuses of the dromedary using CT imaging.

Building on these advances, the present study aims to characterize the brain–skull relationships of the dromedary using 3D CT‐based virtual sections. This approach provides detailed morpho‐topographical insights that are relevant for comparative anatomy, neuroanatomy teaching, and clinical imaging.

## 2. Materials and Methods

### 2.1. Specimen

The study was conducted on the fresh head of a single 18‐month‐old male dromedary camel (*Camelus dromedarius*) of local Saharan type. The animal was slaughtered for human consumption following standard halal procedures in a licensed abattoir. No euthanasia or invasive procedures were performed for research purposes.

After slaughter, the head was carefully disarticulated at the atlanto‐occipital joint, ensuring preservation of cranial integrity. The specimen was immediately placed in a cooled container and transported to the imaging facility for CT examination.

### 2.2. CT Acquisition

CT was performed, using a 16‐slice helical CT scanner (Aquilion Lightning, Canon Medical Systems).

**FIGURE 1:**
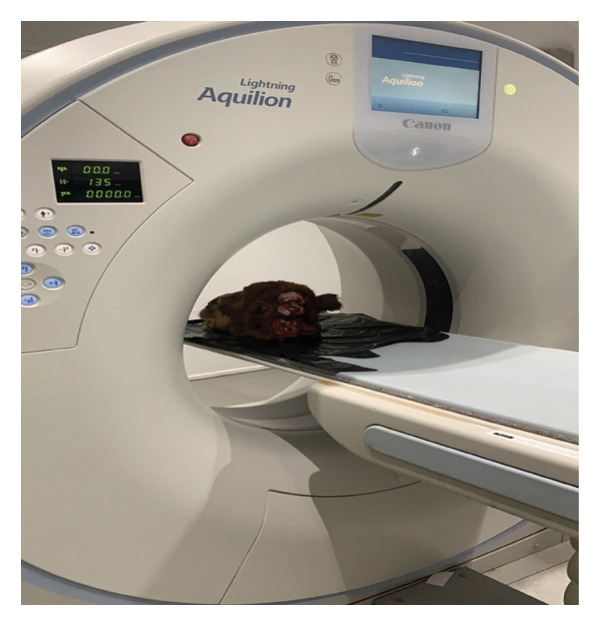
Positioning of the camel head at the CT scan.

The head was positioned in ventral recumbency on the scanning table (Figure 1). Image acquisition was performed primarily in the transverse plane, with subsequent multiplanar reconstructions (sagittal and dorsal) generated digitally.

The CT acquisition parameters were as follows:

• Tube voltage: 120 kV
• Tube current: 200 mA
• Rotation time: 1 s
• Slice thickness: 1 mm
• Reconstruction interval: 0.5 mm
• Bone window: 2500/450 HU
• Soft‐tissue window: 350/40 HU

Images were reconstructed using standard bone and soft‐tissue algorithms and exported in DICOM format for further analysis.

### 2.3. Image Processing and 3D Reconstruction

3D reconstructions and virtual anatomical sections were generated using the open‐source software 3D Slicer.

Multiplanar reconstructions (axial, sagittal, and dorsal planes) were obtained from the DICOM dataset. Anatomical structures were identified using a manual and semiautomatic segmentation approach, based on grayscale contrast differences between bone, soft tissues, and intracranial structures. No automated atlas‐based segmentation was applied.

Segmentation focused on

• Cranial bones
• Paranasal sinuses
• Major brain regions
• Ventricular system
• Vascular and sinus spaces

Surface smoothing and visualization adjustments were applied solely for educational and anatomical clarity, without altering anatomical boundaries.

### 2.4. Anatomical Identification and Figure Preparation

Anatomical identification was performed by comparative analysis with classical and contemporary veterinary anatomy references, following the Nomina Anatomica Veterinaria (NAV) terminology.

Reconstructed images were exported as high‐resolution JPEG files. Minor contrast enhancement and layout adjustments were carried out using Adobe Photoshop CS6, followed by labeling and figure assembly using Microsoft PowerPoint.

Figure annotations were carefully verified to ensure terminological consistency and anatomical accuracy across all planes and section levels.

### 2.5. Study Design and Limitations

This study was designed as a descriptive, atlas‐like anatomical investigation, based on a single representative specimen. The objective was not to perform morphometric or statistical analysis, but to illustrate spatial brain–skull relationships through virtual sections, with an emphasis on anatomical teaching, comparative anatomy, and clinical imaging interpretation.

## 3. Results

### 3.1. Rostral Region: Olfactory Bulb and Frontal Sinuses

**FIGURE 2:**
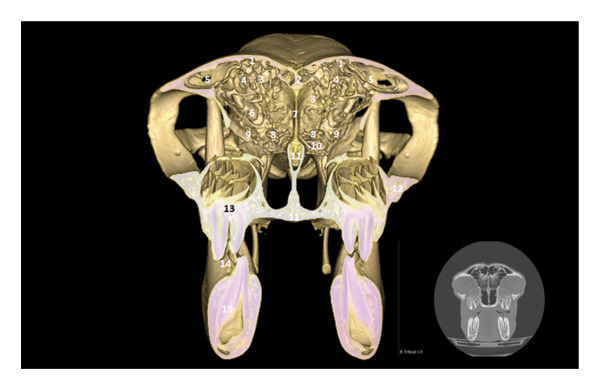
3D reconstruction and CT cross section of the head of a dromedary passing through the olfactory bulb by 3D reconstruction. 1: frontal bone; 2: medial septum of the frontal sinuses; 3: caudal frontal sinus; 4: medial frontal sinus; 5: lateral frontal sinus; 6: olfactory bulb; 7: perpendicular lamina of the ethmoid bone; 8: endoturbinal volutes of the ethmoid bone; 9: ectoturbinal volutes of the ethmoid bone; 10: papery lamina; 11: bone vomer; 12: zygomatic process of the temporal bone; 13: upper third molars M3; 14: diverticulum of the palate; 15: lower third molar M3.

The 3D reconstruction shows the olfactory bulb located at the rostral part of the cranial cavity, in a ventral position with respect to the frontal sinuses (Figure 2). It rests on the riddled lamina of the ethmoid bone, represented here by the perpendicular lamina. The endoturbinal and ectoturbinal volutes testify to the complexity of the ethmoid labyrinth, involved in the conditioning of the inspired air. The median septum separates the two frontal compartments.

### 3.2. Chiasmatic Region: Visual Pathways and Skull Base

**FIGURE 3:**
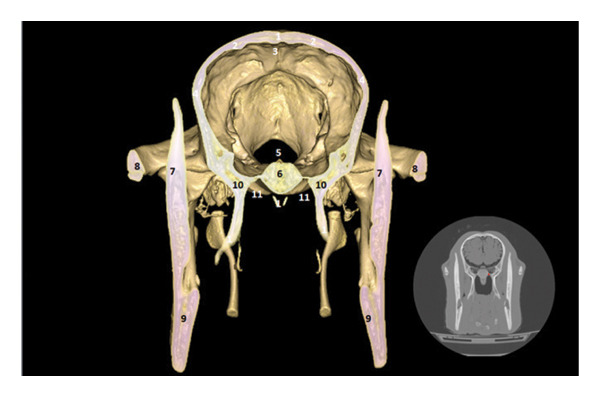
3D reconstruction and CT cross section of a dromedary head through the optic chiasm by 3D reconstruction. 1: external sagittal crest; 2: frontal bone; 3: dorsal sagittal sinus; 4: parietal bone; 5: interthalamic adhesion; 6: body of the bone basisphenoid; 7: branch of the mandible 8: zygomatic process of the temporal bone; 9: inferior alveolar nerves zone; 10: large wing of the sphenoid bone; 11: vein, artery, and maxillary nerve area.

The optic chiasm is located above the body of the basisphenoid bone, in front of the interthalamic adhesion (Figure 3). The sphenoid bone is well represented by its large wing, which participates in the lateral wall of the cranial cavity. The maxillary vasculo‐nerve structures are visible laterally.

### 3.3. Pituitary Region: Pituitary Fossa and Cavernous Sinus

**FIGURE 4:**
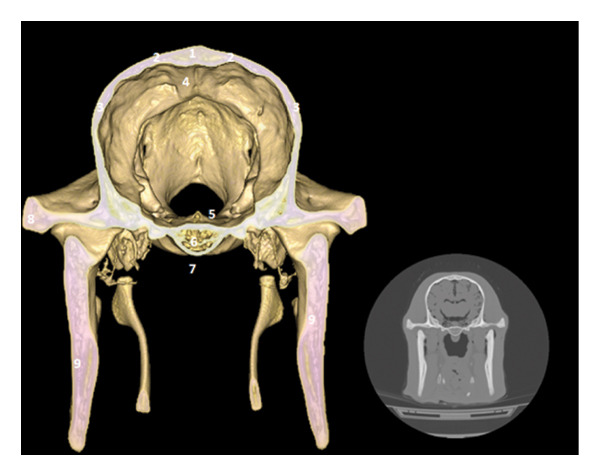
3D reconstruction and CT cross section through the pituitary gland by 3D reconstruction. 1: external sagittal crest; 2: parietal bone; 3: temporal bone; 4: cerebral longitudinal fissure; 5: cavernous sinus; 6: body of the bone basisphenoid; 7: sphenoid crest; 8: zygomatic process of the temporal bone; 9: branch of the mandible.

The pituitary gland, which can be located between the cavernous sinuses, is located in the pituitary fossa, dug into the basisphenoid. The temporal bones participate in the lateral wall, and the cerebral longitudinal fissure is well marked in the upper view (Figure 4).

### 3.4. Peduncular and Cerebellar Region

**FIGURE 5:**
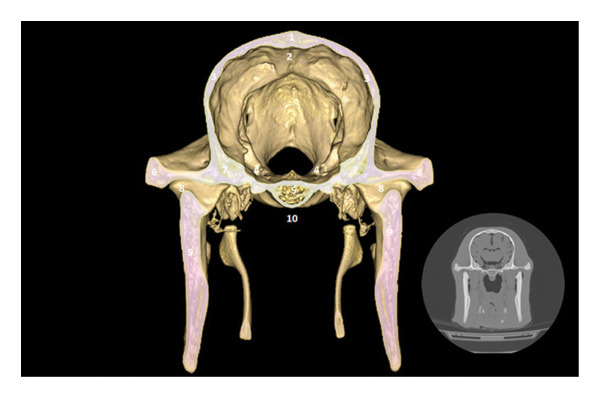
3D reconstruction and CT cross section of a dromedary head through the cerebral peduncle by 3D reconstruction. 1: external sagittal crest; 2: dorsal sagittal sinus; 3: parietal bone; 4: cavernous sinus; 5: bone basisphenoid; 6: zygomatic process of the temporal bone; 7: temporal bone; 8: temporomandibular joint; 9: branch of the mandible; 10: sphenoid crest.

**FIGURE 6:**
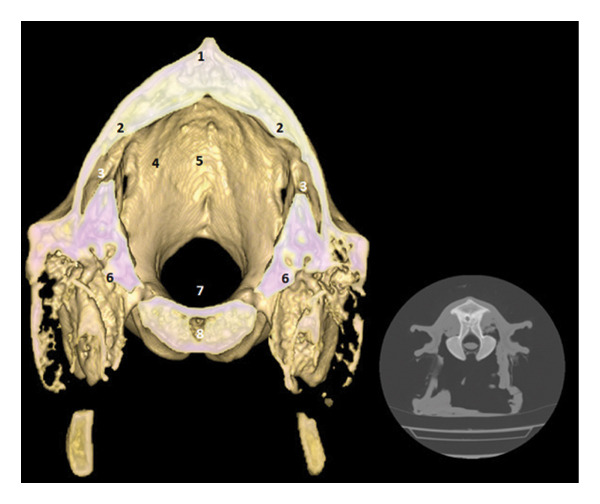
3D reconstruction and CT cross section of a dromedary head through the cerebellum by 3D reconstruction. 1: external sagittal crest; 2: parietal bone; 3: dorsal venous sinus; 4: subarachnoid space; 5: folium of the vermis; 6: petrous part of the temporal bone; 7: medullary pyramids; 8: basioccipital bone.

The cerebral peduncles and cerebellum are located in the caudal portion of the skull. The cerebellum is housed in the cerebellar fossa, under the protection of the parietal bone and the basioccipital bone. The subarachnoid space and the dorsal venous sinus are identifiable (Figures 5 and 6).

### 3.5. Median Sagittal Sections: Cranial Floor and Roof

**FIGURE 7:**
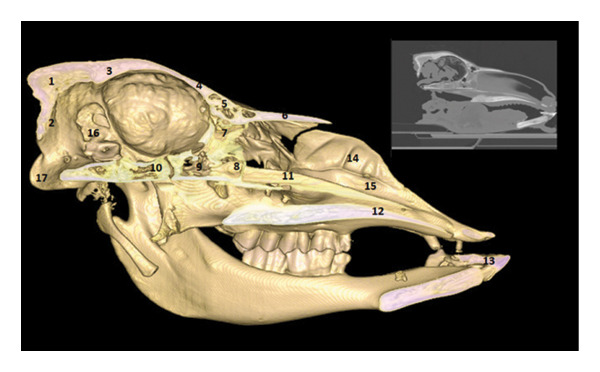
3D reconstruction and CT sagittal section of a camel’s head passing through the median plane by 3D reconstruction. 1: occipital bone; 2: occipital condyle; 3: parietal bone; 4: frontal bone; 5: frontal sinus; 6: nasal bone; 7: ethmoid bone; 8: sphenoid sinus; 9: bone basisphenoid; 10: basioccipital bone; 11: nasal septum; 12: hard palate; 13: lower incisor; 14: ventral nasal concha; 15: ventral meatus of the nasal cavity; 16: cerebellar fossa; 17: foramen magnum.

**FIGURE 8:**
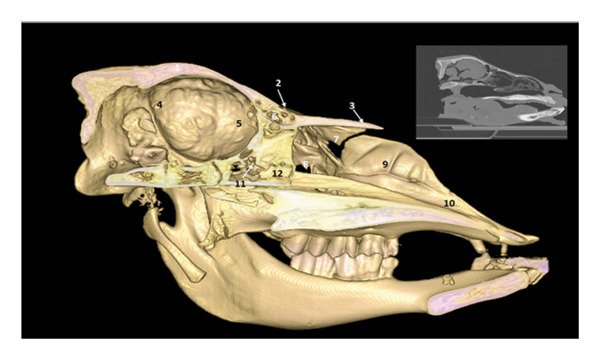
3D reconstruction and CT sagittal section of the head of a dromedary passing through the median plane by 3D reconstruction. 1: ventral part of the ventral nasal turbinate; 2: dorsal part of the ventral nasal turbinate; 3: nasal bone; 4: nasal septum; 5: maxillary bone; 6: middle conchal sinus; 7: dorsal conchal sinus; 8: maxillary sinus; 9: maxillary bone; 10: bone vomer; 11: eyeball; 12: zygomatic process of the frontal bone.

**FIGURE 9:**
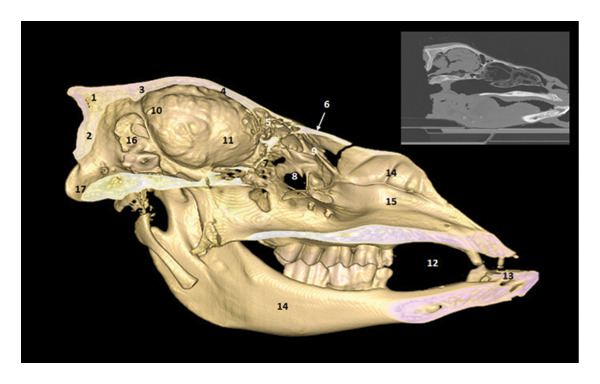
3D reconstruction and CT sagittal section of the head of a dromedary passing through the median plane by 3D reconstruction. 1: occipital bone; 2: occipital condyle; 3: parietal bone; 4: frontal bone; 5: frontal sinus; 6: nasal bone; 7: ethmoid bone; 8: maxillary sinus; 9: dorsal conchal sinus; 10: dorsal septum; 11: maxillary bone; 12: oral cavity; 13: lower incisor; 14: ventral nasal concha; 15: ventral meatus of the nasal cavity; 16: cerebellar fossa; 17: foramen magnum.

**FIGURE 10:**
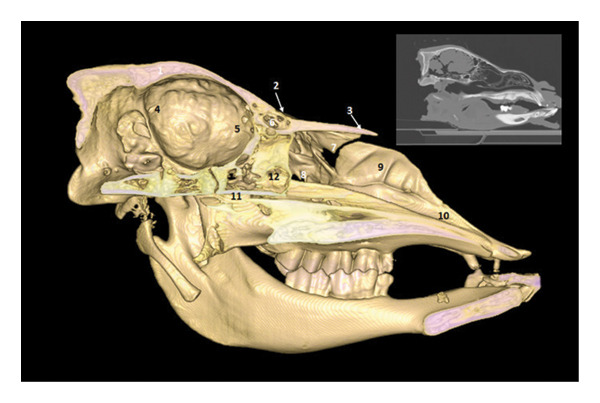
Sagittal section of a camel’s head passing through the median plane by 3D reconstruction and CT cross section. 1: ventral part of the ventral nasal turbinate; 2: dorsal part of the ventral nasal turbinate; 3: nasal bone; 4: nasal septum; 5: maxillary bone; 6: middle conchal sinus; 7: dorsal conchal sinus; 8: maxillary sinus; 9: maxillary bone; 10: bone vomer; 11: eyeball; 12: zygomatic process of the frontal bone.

**FIGURE 11:**
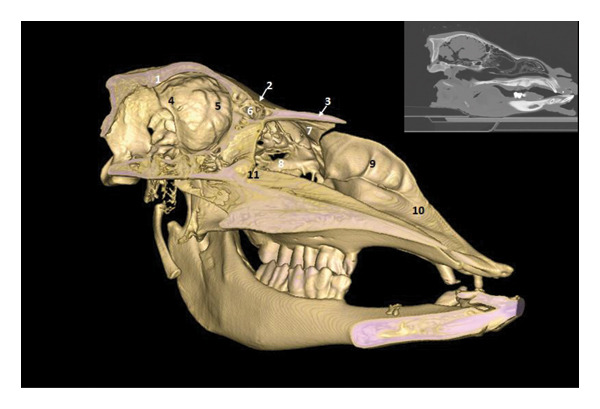
3D reconstruction and CT sagittal section of the head of a dromedary passing through the median plane with a degree of inclination of 30° by 3D reconstruction. 1: ventral part of the ventral nasal turbinate; 2: dorsal part of the ventral nasal turbinate; 3: nasal bone; 4: nasal septum; 5: maxillary bone; 6: middle conchal sinus; 7: dorsal conchal sinus; 8: maxillary sinus; 9: maxillary bone; 10: bone vomer; 11: zygomatic process of the frontal bone.

**FIGURE 12:**
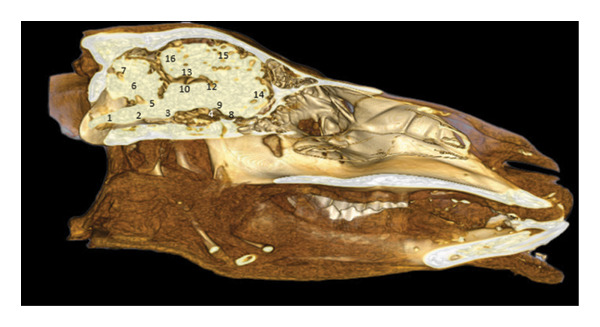
3D reconstruction and CT cross section of the dromedary head (sagittal section). 1: spinal cord; 2: medulla oblongata; 3: pons; 4: pituitary gland; 5: fourth ventricle; 6: medullary body of the cerebellum; 7: cerebellum; 8: optic chiasm; 9: third ventricle; 10: interthalamic adhesion; 11–13: corpus callosum; 11: knee; 12: trunk; 13: splenium; 14: frontal lobe; 15: parietal lobe; 16: occipital lobe.

**FIGURE 13:**
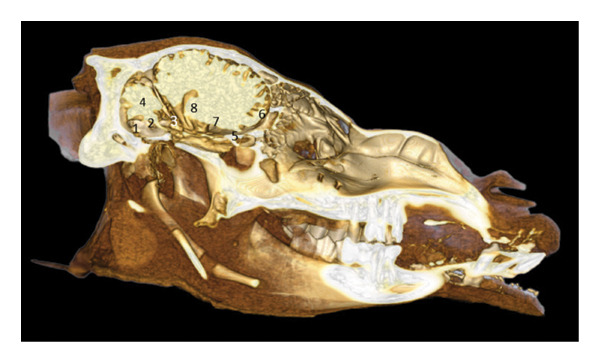
3D reconstruction and CT cross section of the camel’s head (sagittal section passing through PM1). 1: medulla oblongata; 2: bridge; 3: pineal gland; 4: fourth ventricle; 5: cerebellum; 6: optic chiasm; 7: third ventricle; 8: thalamus; 9: olfactory bulb.

**FIGURE 14:**
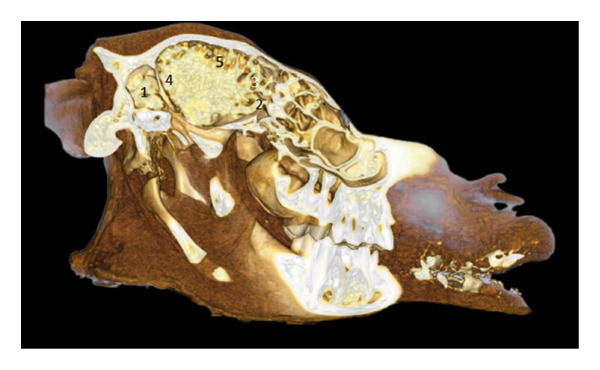
3D reconstruction and CT cross section of the dromedary head (sagittal section passing through M2). 1: cerebellum; 2: olfactory bulb; 3: frontal lobe; 4: occipital lobe; 5: parietal lobe.

All the cerebral lobes are represented: frontal, parietal, and occipital. The corpus callosum is visible with its different parts: knee, trunk, and splenium. Diencephalic structures, such as the thalamus, hypothalamus, and pineal gland, are highlighted. The position of the medulla oblongata and the pons makes it possible to understand the anchoring of the brainstem to the cranial floor. The fourth ventricle, the horns of Ammon, and the lateral ventricles illustrate the cerebral ventricular system (Figures 7, 8, 9, 10, 11, 12, 13, and 14).

### 3.6. Lateral and Dorsal Views of the Brain in Place

**FIGURE 15:**
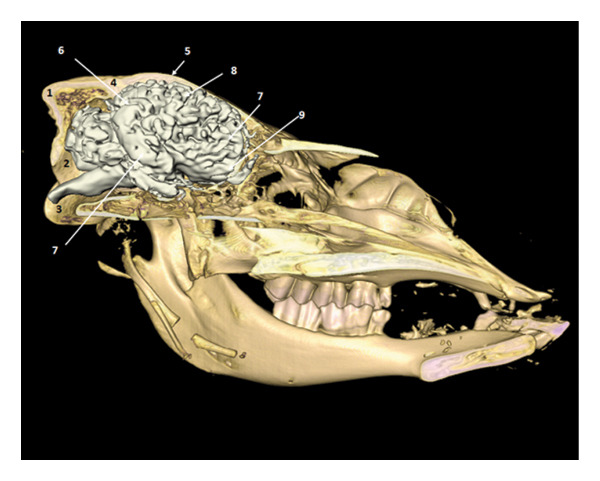
3D reconstruction and CT cross section of the brain in place (right half of the skull removed) (side view). 1: occipital bone; 2: occipital condyle; 3: foramen magnum; 4: parietal bone; 5: frontal bone; 6: occipital lobe; 7: temporal lobe; 8: parietal lobe; 9: olfactory bulb.

**FIGURE 16:**
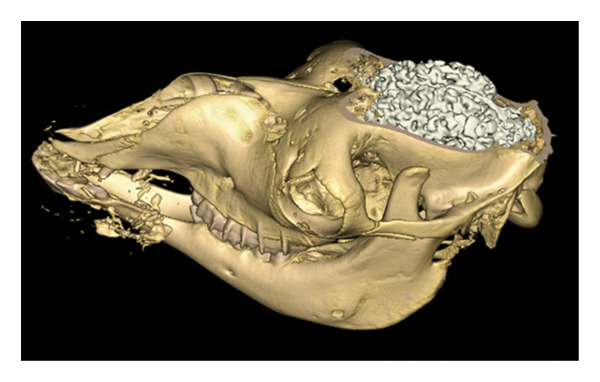
Dorsal view of the brain in situ after removal of the dorsal half of the skull: rostral cerebral lobes and olfactory region.

**FIGURE 17:**
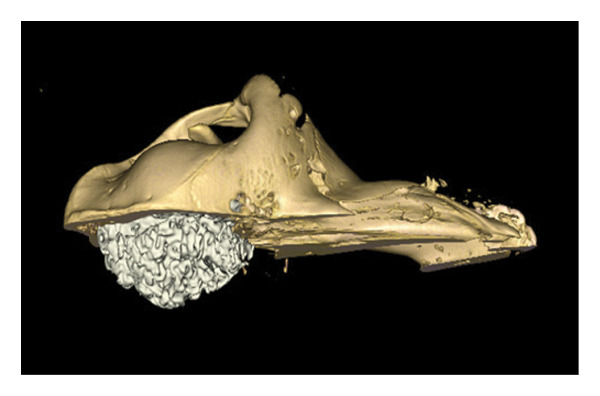
Dorsal view of the brain in situ after removal of the dorsal half of the skull: caudal cerebral lobes and cerebellar region.

After removal of the right or dorsal half of the skull, the cerebral lobes (frontal, temporal, parietal, and occipital) appear in natural projection into the cranial cavity. The olfactory lobe is well individualized in the rostral position. The longitudinal cerebral fissure separates the two hemispheres, facilitating the identification of the cerebral cortex (Figures 15, 16, and 17).

### 3.7. Regional Cross Sections

**FIGURE 18:**
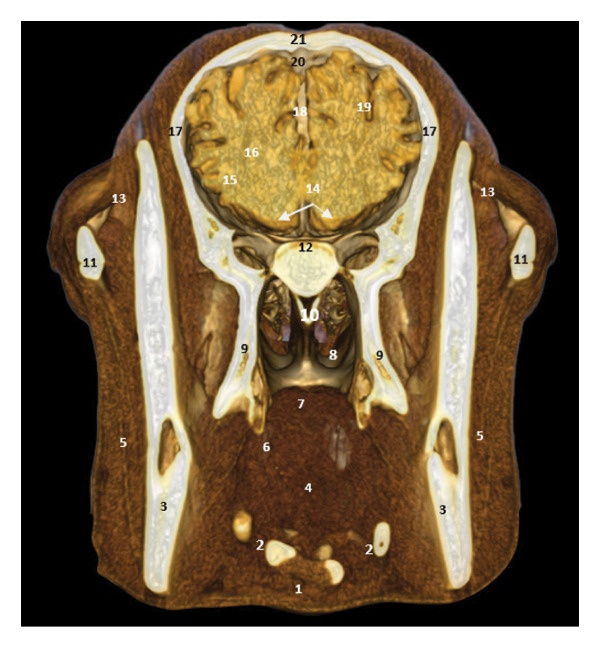
3D reconstruction and CT cross section of the dromedary head (cross section of the head passing through the rostral half of upper PM2). 1: geniohyoid and mylohyoid muscle; 2: artery of the tongue; 3: mandibular bone; 4: language base; 5: masseter muscle; 6: oropharynx; 7: soft palate; 8: nasopharynx; 9: medial pterygoid muscle; 10: nasal septum; 11: zygomatic process of the temporal bone; 12: velvety of the ethmoid bone; 13: infraorbital adipose bodies; 14: optic chiasm; 15: gray matter; 16: white matter; 17: temporal bone; 18: longitudinal cerebral fissure; 19: cerebral cortex (frontal lobe); 20: sagittal dorsal sinus; 21: frontal bone.

**FIGURE 19:**
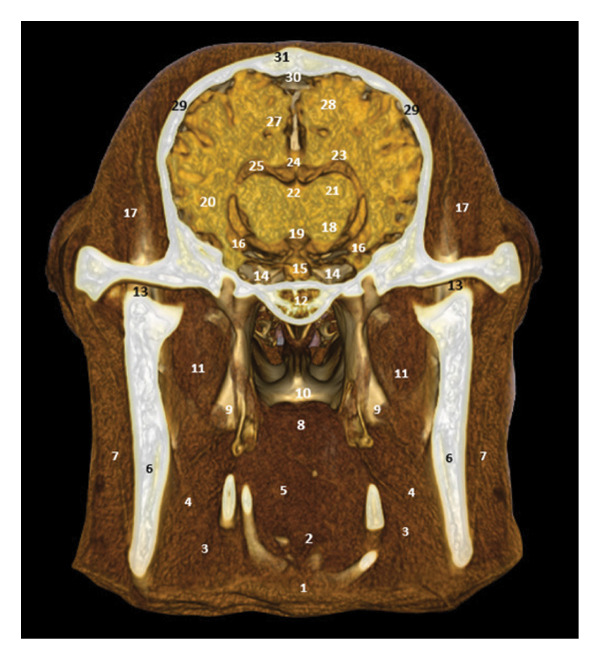
3D reconstruction and CT cross section of the dromedary head (cross section of the head passing through the ascending branch of the mandible). 1: omohyoid and thyroid muscle; 2: language base; 3: horn of the hyoid bone; 4: pharyngeal muscle; 5: oropharynx; 6: ascending branch of the mandible; 7: masseter muscle; 8: soft palate; 9: medial pterygoid muscle; 10: nasopharynx; 11: lateral pterygoid muscle; 12: base of the sphenoid bone; 13: temporomandibular disc; 14: cavernous sinus; 15: pituitary gland; 16: horns of Ammon; 17: temporal muscle; 18: hypothalamus; 19: third ventricle; 20: cerebral cortex; 21: thalamus; 22: body of the fornix; 23: striated body; 24: corpus callosum; 25: lateral ventricle; 26: white matter; 27: gyrus cingulum; 28: sagittal gyrus; 29: temporal bone; 30: sagittal dorsal sinus; 31: parietal bone.

**FIGURE 20:**
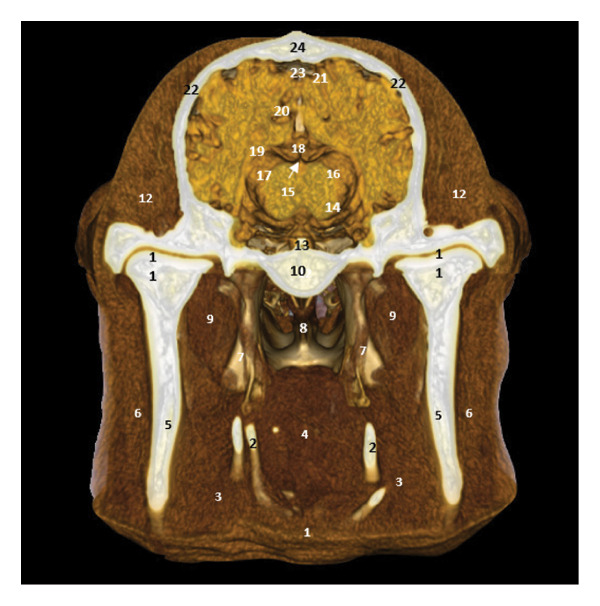
3D reconstruction and CT cross section of the dromedary’s head (cross section of the head passing through the middle of the temporomandibular joint). 1: esophagus; 2: hyoid bone; 3: pharyngeal muscle; 4: soft palate; 5: ascending branch of the mandible; 6: masseter muscle; 7: nerves V; 8: nasopharynx; 9: lateral pterygoid muscle; 10: basisphenoid bone; 11: temporomandibular disc; 12: temporal muscle; 13: pituitary gland; 14: hypothalamus; 15: Body of the fornix; 16: thalamus; 17: horn of Ammon; 18: corpus callosum; 19: lateral ventricle; 20: gyrus cingulum; 21: sagittal gyrus; 22: temporal bone; 23: sagittal dorsal sinus; 24: parietal bone.

These sections show the camel’s head at different levels. Figure 18, at the level of the upper PM2, highlights the oropharyngeal structures, the optic chiasm, and the cerebral lobes. Figure 19, at the level of the ascending branch of the mandible, reveals the pituitary gland, the thalamus, and the corpus callosum. Figure 20, at the temporomandibular joint, shows the horn of Ammon, the fornix, the cerebral gyrus, and the lateral ventricles (Figures 18, 19, and 20).

### 3.8. Posterior Dorsal Sections

**FIGURE 21:**
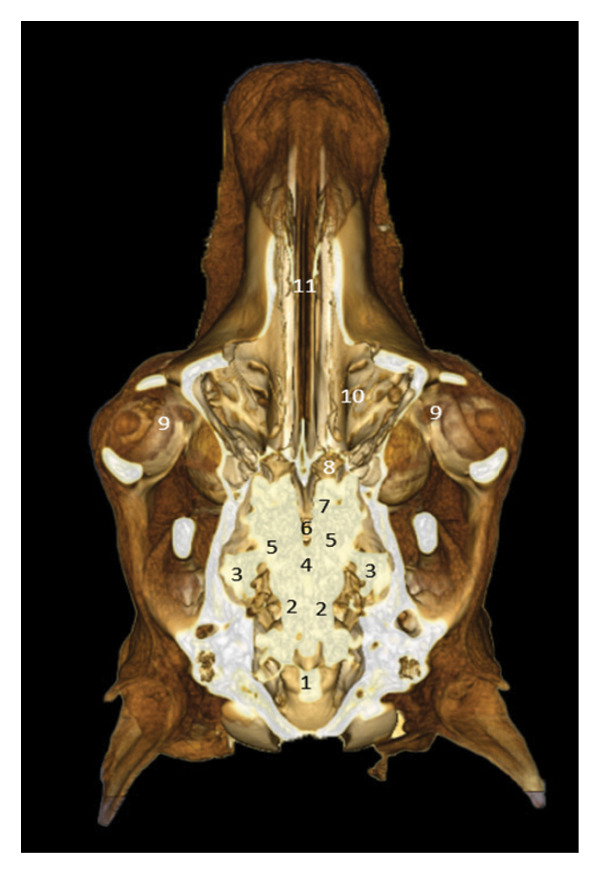
3D reconstruction and CT cross section of the camel’s head (dorsal section passing through the bulb of the eye). 1: cerebellum; 2: rostral cerebellar peduncle; 3: flocculus; 4: fourth ventricle; 5: thalamus; 6: third ventricle; 7: caudate nucleus; 8: olfactory bulb; 9: infraorbital adipose body; 10: velvety ethmoid bone; 11: nasal bone.

**FIGURE 22:**
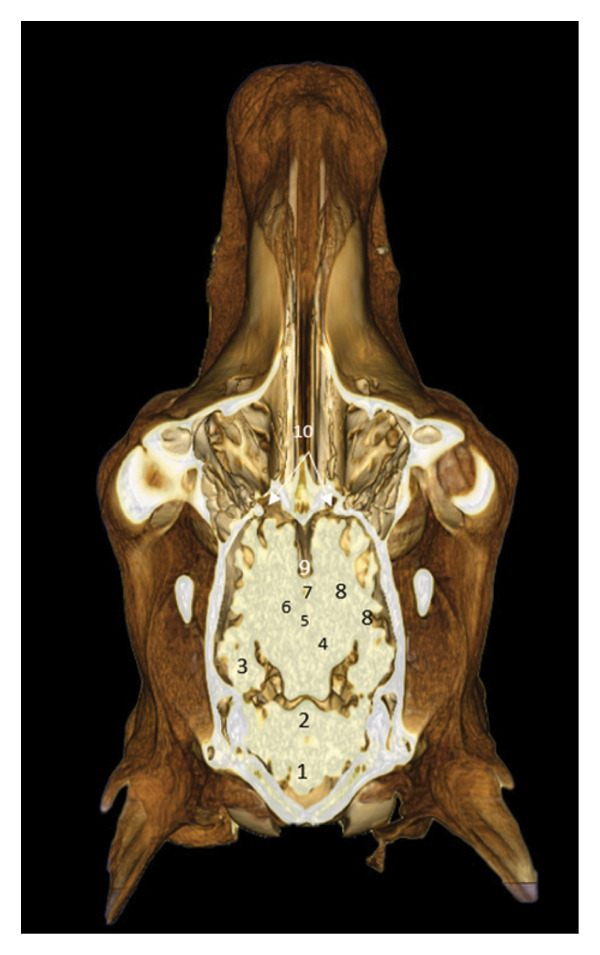
3D reconstruction and CT cross section of the camel’s head (dorsal section passing through the lacrimal sinus). 1: cerebellum; 2: rostral cerebellar peduncle; 3: horn of Ammon; 4: thalamus; 5: body of the fornix; 6: striated body; 7: lateral ventricle; 8: internal capsule; 9: longitudinal cerebral fissure; 10: olfactory bulb.

**FIGURE 23:**
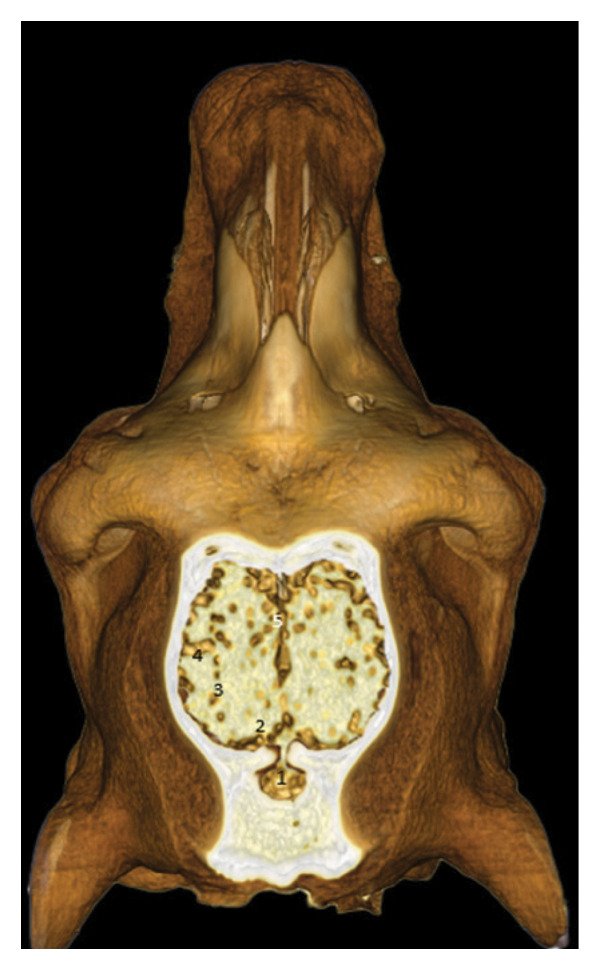
3D reconstruction and CT cross section of the dromedary head (dorsal section passing through the parietal bone). 1: cerebellum; 2: occipital gyrus; 3: ectomarginal gyrus; 4: middle ectosylvian gyrus; 5: longitudinal cerebral fissure.

These views show the posterior relationships of the brain, with the cerebellum, cerebellar peduncles, thalamus, internal capsule, and olfactory lobes. Cortical gyri are identified: occipital, ectomarginal, and ectosylvian gyri (Figures 21, 22, and 23).

## 4. Discussion

The present study provides a 3D CT‐based descriptive analysis of the spatial relationships between the brain and the skull in the dromedary camel (*Camelus dromedarius*), using virtual anatomical sections generated from a single representative specimen. The approach adopted is intentionally descriptive and atlas‐oriented, aiming to support anatomical teaching, comparative anatomy, and clinical imaging interpretation rather than population‐level inference.

The cross sections illustrate the central position of the well‐developed olfactory bulb located in the rostral region of the skull. This arrangement confirms the importance of the dromedary olfactory system, already described by Adah and al. [5], as essential for the detection of water, congeners, and food. In addition, the richness and complexity of ethmoid turbinals (endoturbinals and ectoturbinals) suggest a filtration and conditioning capacity for inspired air, which is essential in desert environments [6].

The configuration of the paranasal sinuses—in particular the frontal, conchal, and maxillary sinuses—shows significant and compartmentalized extensions. These sinuses act as resonance cavities, areas of weight reduction of the skull, and thermoregulatory devices [7]. Their topographical relationship with the cerebral lobes and nasal cavities reinforces the idea of an optimized functional arrangement.

Sagittal and dorsal reconstructions of the brain in situ allow for a clear visualization of the compartmentalization of the cerebral lobes, in particular the frontal, parietal, temporal, and occipital lobes. This partitioning is consistent with observations made on other ruminants [8], but the proportions in the dromedary seem to have changed slightly, particularly at the level of the more prominent olfactory lobe.

Our results detail the intracranial anatomy: optic chiasm, pituitary gland, thalamus, cerebral ventricles, and commissural formations (corpus callosum and fornix). Their position shows a rostrocaudal orientation close to that described in cattle and equines [9], with however a certain apparent compactness, which could reflect an adaptation to thermal resistance and mechanical stress [10].

The richness of the images obtained also makes it possible to study the interactions between masticatory, respiratory, and encephalic structures. The embedding of the mandible, the development of the masseter muscle, and the presence of venous sinuses show the importance of muscle support and intracranial venous circulation in the physiological adaptation of the dromedary [11].

In addition, although similar anatomical features of the dromedary head have been previously described [4], the present study contributes complementary information by focusing specifically on brain–skull spatial relationships. We emphasize that each 3D reconstruction generated in this study provides a valuable didactic opportunity. When analyzed individually, these virtual sections can serve as pedagogical support for teaching veterinary neuroanatomy, facilitating the understanding of complex 3D relationships that are not easily accessible through classical dissection or 2D imaging. This educational dimension reinforces the relevance of 3D reconstruction tools in veterinary training.

This study is limited by the use of a single specimen, which precludes assessment of interindividual variability related to age, sex, or breed. Consequently, the findings should be regarded as illustrative rather than representative of the entire species. Future studies involving multiple specimens and complementary morphometric analyses would be required to extend these observations and support quantitative comparisons.

## 5. Conclusion

3D CT‐based virtual anatomical sections of the dromedary head provide a detailed and coherent visualization of brain–skull relationships. The reconstructions illustrate the close spatial integration of neuroanatomical structures, cranial bones, and paranasal sinuses, reflecting functional organization adapted to the specific biological context of this species.

Although based on a single specimen, this atlas‐style descriptive study demonstrates the value of CT‐derived 3D reconstructions as a noninvasive tool for exploring complex anatomical relationships. The results contribute a useful visual reference for veterinary education, comparative neuroanatomy, and clinical imaging of camelids, while highlighting the need for future studies incorporating larger sample sizes to explore anatomical variability.

## Author Contributions

This work was carried out in collaboration among all authors.

## Funding

The authors received no specific funding for this work.

## Disclosure

All authors have read and approved the final manuscript.

## Ethics Statement

No live animal procedures were performed in this study. The specimen consisted of the head of a dromedary camel obtained postmortem from a licensed abattoir, where the animal had been slaughtered for human consumption following standard procedures. According to institutional and national regulations, the use of postmortem abattoir material for anatomical research is exempt from ethical committee approval. All handling and imaging procedures complied with applicable guidelines.

## Conflicts of Interest

The authors declare no conflicts of interest.

## Data Availability

The data that support the findings of this study are available from the corresponding author upon reasonable request.
